# Society Position Statements on Bio-Identical Hormones-Misinformation Leads to a Dilemma in Women’s Health

**DOI:** 10.3390/healthcare9070782

**Published:** 2021-06-22

**Authors:** Gary S. Donovitz

**Affiliations:** 1 Department of Obstetrics and Gynecology, Morehouse School of Medicine, Atlanta, GA 30310, USA; gary@donovitz.com; 2 BioTE Medical LLC, Irving, TX 75038, USA

**Keywords:** hormone replacement therapy, bio-identical hormones, compounded bio-identical hormones

## Abstract

This commentary reviews the current status of compounding pharmacies and underscores outdated and inaccurate information in the clinical opinions and position statements of two prominent societies.

## 1. History

There has been limited and outdated guidance from both The American College of Obstetrics and Gynecology (ACOG) and The North American Menopause Society (NAMS) regarding the utilization of bio-identical hormones in women. Prior to the Women’s Health Initiative (WHI), hormone replacement therapy was used to treat the symptoms of menopause and improve the overall quality of life and health of women worldwide. In fact, in a randomized controlled trial, there were improvement in the somatic and psychological complaints affecting post-menopausal women [1,2].

Following the WHI, which reported an increased risk of cardiovascular disease, invasive breast cancer, venous thromboembolism, and stroke associated with the use of the single drug PremPro^®^ (an oral combination synthetic hormone pill) (Pfizer Inc., New York, NY, USA), the indications and utilization of Hormone Replacement Therapy (HRT) have changed dramatically. Many physicians, fearing reported side effects, have stopped prescribing HRT. Moreover, many patients have retreated to the sidelines, feeling that no HRT may be the safer alternative. *Time* magazine dramatized the outcome further by stating that hormone replacement therapy was “riskier than advertised” [3].

Thus, treatment for longevity and long-term quality of life are no longer indications for menopausal hormone therapy. This has limited HRT to short-term treatment of menopausal symptoms. The reduction in estrogen replacement therapy that occurred after the WHI has led to tens of thousands of deaths in women according to one study [4]. The North American Menopause Society reports that nearly 2 million women enter menopause yearly and that 40% of menopause patients are using bio-identical hormones.

## 2. Published Society Opinions

The ACOG Committee Opinion number 322 (2005), [5] Committee Opinion 387 (2007) [6], and Committee Opinion 532 (2012) [7] titled Compounded Bioidentical Menopause Therapy has stated that:

*“Not only is evidence lacking to support superiority claims of compounded bioidentical hormones over conventional menopausal hormone therapy, but these claims also pose the additional risks of variable purity and potency and lack efficacy and safety data.”*

In 2017 [8], the North American Menopause Society published its Position Statement which included the following excerpts:

*“Compounded hormone therapies are prepared by a compounding pharmacist using a provider’s prescription and may combine multiple hormones (estradiol, estrone, estriol, dehydroepiandrosterone [DHEA], testosterone, progesterone), use untested, unapproved combinations or formulations, or be administered in nonstandard (untested) routes such as subdermal implants, pellets, or troches.”*

*“Compounded HT has been prescribed or dosed on the basis of salivary hormone testing; however, salivary testing for HT is considered unreliable because of differences in hormone pharmacokinetics and absorption, diurnal variation, and interindividual and intraindividual variability.”*

*“Prescribers should only consider compounded HT if women cannot tolerate a government-approved therapy for reasons such as allergies to ingredients or for a dose or formulation not currently available in government-approved therapies. With interim guidance on compounding safety and quality control from FDA, quality control of compounded HT may improve.”*

*“Compounded bioidentical HT presents safety concerns such as minimal government regulation and monitoring, overdosing or underdosing, presence of impurities or lack of sterility, lack of scientific efficacy and safety data, and lack of a label outlining risks.”*

As these are the most updated statements and opinions, they remain outdated and are not accurate in depicting the current state of compounded bio-identical hormone therapy.

## 3. Compounding

Just as pharmaceutical manufacturing has evolved and improved over the past decades, the same can be said for compounding. In a world where HRT has been more established for women, the availability of individualized dosing of hormones has relied heavily on compounding pharmacies to achieve products of the greatest need for women’s health. Individualized dosing more precisely addresses the hormone deficiencies of each patient and contributes to greater benefits while simultaneously minimizing risk. This is especially true of testosterone where there are no commercially available products for practitioners to prescribe for women.

“Compounding is the creation of an individualized preparation in response to a health care provider’s prescription to create a medication tailored to the specialized needs of an individual patient“, according to the Position Statement (532) published by the ACOG. This definition is both antiquated and incomplete because of The Drug Quality and Security Act in 2013, which added a new section 503B to the FD&C Act [9]. Under section 58 503B(b), a compounder can register as an outsourcing facility with the FDA. Drug products compounded in an outsourcing facility can qualify for exemptions from the FDA approval requirements if the conditions in section 503B are met. Outsourcing facilities are inspected by the FDA and must comply with other provisions of the FD&C Act, including CGMP (current good manufacturing practice) requirements under section 65 501(a)(2)(B). Furthermore, the term “current good manufacturing practice” includes the implementation of oversight and controls over the manufacture of drugs to ensure quality, which includes “managing the risk of and establishing the safety of raw materials, materials used in the manufacturing of drugs, and finished drug products.” This also includes the testing for purity, potency, and (where appropriate) the sterility of the final compounded products prior to their shipping to either physicians’ offices or patients. The FDA felt quality is best assured by implementing appropriate controls throughout the manufacturing process, with end-product testing providing additional assurance. Accordingly, this guidance focuses on the control of raw materials, facility design and maintenance, production techniques and controls, and personnel practices as the most critical aspects of ensuring quality for all drug products. These controls are as rigorous as those imposed on pharmaceutical manufacturers.

Additionally, they are required to report serious and unexpected side effects and complications from their products directly to the FDA, which adds a layer of safety on a foregoing basis regarding adverse outcomes that might occur from their products.

## 4. Women’s Health and Wellness

Healthcare has entered an era of precision medicine. Better stated, we have graduated to individualized healthcare and, as such, women very much want to co-pilot their care. Furthermore, women are trying to feel their best no matter their age. Thus, longevity with vitality matters. They want to avoid many of the diseases suffered by their ancestors such as Alzheimer’s disease, cardiovascular disease, osteoporosis, and breast cancer. The slogan to take hormones in the lowest dose possible for the shortest time possible should be phased out based on newer evidence in the existing literature [10].

Many of the symptoms in both pre-menopause and post-menopause have been successfully treated with bio-identical hormones (both commercially available and compounded). These symptoms include hot flashes, night sweats, insomnia, mood disorders, fatigue, joint pains, and many others. The response to these hormones has been reported in multiple citations in the existing literature [11,12,13,14]. Whereas there is a paucity of data regarding many of the compounded cream formulations, the literature has substantial support for subdermal implants and their benefits for the past 80 years.

The position statement from the North American Menopause Society (NAMS) has focused on the unreliability of salivary testing but has failed to mention the reliability of serum hormone testing, particularly concerning the use of RIA (radioimmunoassay) and HPLC (High-performance liquid chromatography) [15].

The statement from the ACOG [16] that “evidence lacking to support superiority claims of compounded bioidentical hormones over conventional menopausal hormone therapy” is unfounded and outdated based on multiple studies. Silverman et al. found that 17 β Estradiol was three standard deviations better than conjugated equine estrogen with or without synthetic progestin [17]. In addition, bioidentical progesterone (micronized) does not increase the risk for DVT and neither does the synthetic counterpart medroxyprogesterone acetate. The non-oral administration of estradiol does not increase the risk of hypercoagulability, whereas the oral synthetic Prempro^®^ does pose that risk [18]. Finally, a landmark finding is that bio-identical testosterone with and without bio-identical estradiol can significantly reduce the risk of breast cancer. Both of these studies have the additional benefit of long-term safety data, which was collected prospectively over 10 years [19,20].

Although the position statement does not mention side effects, side effects do exist even with improvements in formulations and routes of administration. Patients may experience weight gain, acne, additional facial hair, somnolence, and hair thinning. Recently, a paper reported on 1.2 million procedures using testosterone and testosterone with estradiol using pellets administered subcutaneously. Over a seven-year period, there were less than 1% secondary reactions [21]. In addition to the benefits (both long and short term) from HRT, the side effects should also be presented.

A more contemporary position statement is needed from both the ACOG and NAMS. Such statements should provide a more accurate depiction of the current state of compounded Bio-identical hormones. These statements should also note that the stringent conditions imposed by the FDA on 503b outsourced pharmacies have improved the purity, potency, and sterility of these products, comparable to that of commercially available hormone products. The question concerning the long-term safety of bio-identical hormones has been answered through years of experience utilizing estradiol patches (e.g., Vivelle^®^ [Manufactured by: Noven Pharmaceuticals Inc., Miami, FL, USA. Distributed by: Novartis Pharmaceuticals Corporation, East Hanover, NJ, USA] and Climara^®^ [Bayer Healthcare, Montville, NJ, USA]) and sub-cutaneous hormone pellet therapy with testosterone and estradiol.

## Data Availability

Not applicable.
